# Bioluminescent imaging of an oomycete pathogen empowers chemical selections and rational fungicide applications

**DOI:** 10.1186/s13007-025-01374-9

**Published:** 2025-05-07

**Authors:** Han Chen, Jiana Mao, Yujie Fang, Waqas Raza, Zhi Li, Chongyuan Zhang, Yingguang Zhu, Yuanchao Wang, Suomeng Dong

**Affiliations:** 1 State Key Laboratory of Agricultural and Forestry Biosecurity, College of Plant Protection, Nanjing Agricultural University, Nanjing, 210095 China; 2 Jiangsu Key Laboratory of Pesticide Science and Department of Chemistry, College of Sciences, Nanjing Agricultural University, Nanjing, 210095 China

**Keywords:** Bioluminescent imaging, Luciferase, Chemical selections, Quantitative high-throughput screening, Late blight

## Abstract

Fungicides play an indispensable role in ensuring food security. However, rational chemical selection and fungicide precision application guidance remain constrained by the limitations in real-time monitoring of tracking pathogens within plant tissues. In the current study, we generated a genetically stable *Phytophthora infestans* strain (PiLuc) expressing *luciferase* gene, which serves as a dual-mode quantification platform for both in vitro and in vivo throughput screening. Consequently, we designed a 96-well plate high-throughput screening system to assess compounds inhibitory efficacy using PiLuc. Crucially, bioluminescence imaging enabled visualization of PiLuc in potato leaves and tubers during early infection stage, which is invisible to the naked eye. Capitalizing on the semi non-destructive and visual advantages, we developed a system for fungicide bioavailability evaluation and dosage-response assessment in tuber tissues, integrating real-time dynamic monitoring of pathogen. The development of bioluminescent imaging of late blight pathogen establishes an enabling platform for high-throughput fungicide screening while improving the precision bioavailability assessments.

**Supplementary Information:**

The online version contains supplementary material available at 10.1186/s13007-025-01374-9.

## Introduction

Plant pathogens and pests are widely recognized as significant obstacles to food production and supply. It’s estimated that up to 40% of global crop production is compromised due to the impact of plant pathogens and pests [1–3]. The annual economic burden caused by pathogens alone amounts approximately $220 billion [4], whereas pesticide application mitigates 30% to 40% of the total crop losses worldwide and becomes a common and indispensable practice in modern intensified farming [5, 6]. Currently, the expanding human population, emerging fungicide resistance and increasing global climatic extremes necessitate the development of new and improved fungicides applications.

Large-scale screening of compounds and novel formulations remains a labor- intensive and time-consuming task, particularly given the exponentially growing market inventory of new chemical entities [7, 8]. In laboratory experiments, precise pathogen quantification serves as a crucial determinant of fungicide efficacy. However, symptom variability arising from hosts, pathogens, environmental fluctuations, nutrient deficiencies, coupled with subjective assessments of infection area and disease index values, may lead to human errors during data collection, particularly for diseases affecting juicy fruits and thick tuber samples [9–11].

Laboratory-based techniques, such as polymerase chain reaction (PCR), immunofluorescence (IF), enzyme-linked immunosorbent assay (ELISA) and mass spectrometry (MS) [12–14], require specialized expertise in both operation and data analysis [15–17]. Moreover, molecular assays often require destructive sample collection steps, such as DNA extraction, which makes it difficult to collect time-course data on identical samples. Such procedures are not suitable for large-scale quantitative screening and assessment. In summary, a straightforward method has so far been missing.

Imaging technology produces extensive, real-time, and quantitative data sets of pathogen proliferation and colonization. Various imaging techniques and computer vision approaches including spectroscopy and remote sensing tools, have been employed to agricultural surveillance [18, 19]. However, direct pathogen monitoring is still limited. Bioluminescence is the light emitted by living organisms, such as the luciferase from *Photinus pyralis* (firefly) [20, 21]. Bioluminescence imaging has been standardized in mammalian disease models for real-time tracking, and quantification of cells or microbes, significantly enhancing our understanding of disease progression and therapeutic effects [22–24]. Its application to pathogenic bacteria enables in vivo evaluation of antimicrobial treatment and host resistance [22–25]. Bioluminescence imaging technology has been explored in studying animal-fungi interaction systems, including the human-Aspergillus fumigatus and human-*Candida albicans* systems [26–28]. Therefore, we raised the question of whether the expression of luc in filamentous pathogens could refine screening steps and offer informed guidance for fungicide application in agriculture and plant science research.

*Phytophthora infestans* is one of the most devastating agents causing potato late blight (PLB). In the 1840 s, the epidemic of PLB resulted in the “Great Irish famine” leading to the deaths or emigration of two million people. Until now, it is the biggest threat to the potato industry worldwide [29, 30]. Oomycetes were long regarded as lower fungi because of their filamentous growth habit; however, large variations, including sensitivity to conventional fungicides, were observed between oomycetes and various fungi [31–33]. The limited arsenal of oomycete-specific fungicides contrasts sharply with the diverse antifungal agents currently available [34]. The restricted availability of fungicides and the rapid development of chemical agents targeting oomycetes necessitate the implementation of a reliable and robust screening method. Previous research successfully used luciferase as a reporter gene in *P. infestans*, however, luciferase activity was very low, even under a highly constitutive promoter [35]. We therefore pursued the development of an optimized bioreporter leveraging this reporter gene.

In this study, we optimized the *luc* sequence and engineered a genetically stable *luc*-labeled *P. infestans* strain PiLuc without impacting its growth and pathogenicity through *Agrobacterium-mediated* transformation (AMT). We developed imaging technology for quantitative screening and evaluating compounds and new formulations based on PiLuc. Firstly, this method enables in vivo compound high-throughput screening in a 96-well format, which greatly increases the capacity of primary screening. Secondly, we designed the procedures to determine the bioavailability and minimum effective dosage of fungicides on potato tubers. Thus, this approach enables quantitative lead compound prioritization, and is likely to become a transformative tool for rational agrochemical development and fungicide deployment.

## Materials and methods

### Growth conditions for *P. infestans* and potato

The *P. infestans* strain JH19 was isolated from infected tomato in San Diego Country, California in 1982 and kindly provided by Howard S. Judelson lab [36]. JH19 and PiLuc were cultured and kept on rye-sucrose + V8 medium (agar 15 g/L) at 18 ℃. 60 g rye grains were soaked in 800 mL distilled water and then sterilized at 121 ℃ for 30 min. The rye-sucrose + V8 medium was prepared as following: the sterilized rye and water were smashed using a blender, filtered with 4 layers of gauze and the sediment was discarded. The supernatant was combined with 100 mL V8 juice (with 1 g CaCO_3_), 20 g sucrose and 15 g agar, then made up to 1 L and sterilized at 121 ℃ for 30 min. The mycelium samples of JH19 and PiLuc were cultured on PEA liquid medium at 18 ℃ for western blotting. The PEA liquid medium was prepared as following: 120 g pea were soaked in 800 mL distilled water and then sterilized at 121 ℃ for 30 min. The supernatant was collected to prepare 1 L liquid medium and sterilized at 121 ℃ for 30 min. Potatoes were grown in controlled environment growth chambers under long-day conditions (16 h light/8 h dark cycle at 140 μE s^−1^ m^−2^ light intensity) at 22 ℃.

### Codon optimization, construction of plasmids, AMT of *P. infestans*

The original firefly *luc* sequence was from pGL3-Control (Promega, https://www.snapgene.com/plasmids/luciferase_vectors/pGL3-Control). The sequence fused with FLAG-tag (DYKDDDDK) was optimized by Sangon Biotech (Shanghai) Co., Ltd (Fig s1a). Briefly, a 1653 bp DNA sequence was optimized for better protein expression in *P. infestans*. Codon usage was adjusted to match the highest expression profile of *P. infestans*, CAI (Codon Adaptation Index) was upgraded from 0.59 to 0.93. (A CAI of 0.8–1.0 is regarded as good for high expression). Additionally, average GC content was adjusted from 46.8% to 64.5% and unfavorable peaks were removed. Once optimized, the sequence was synthesized and cloned into a pUC57 vector (https://www.snapgene.com/plasmids/basic_cloning_vectors/pUC57). The *luc-flag* sequence was PCR-amplified and cloned into the I-CeuI site of the pLY40 vector [37] using the ClonExpress II One Step Cloning Kit (Vazyme, C112). Following successful cloning, the plasmid was transformed into *Escherichia coli* strain JM109 and selected on 50 μg/mL ampicillin-containing LB medium. Subsequently, the plasmid of correct JM109 colony was extracted and transformed into *Agrobacterium tumefaciens* strain EHA105 with helper plasmid pV1F [37]. The colonies were selected on 25 μg/mL rifampicin, 50 μg/mL kanamycin and 100 μg/mL spectinomycin-containing LB medium.

The AMT of *P. infestans* zoospores was conducted following published methods [37, 38]. In brief, *A. tumefaciens* strain EHA105 was cultured at room temperature for 1 h by MS liquid medium with 200 mM acetosyringone, and then mixed with about 10^5^ *P. infestans* zoospores at room temperature for 1 h. The zoospores were then collected and spread at Whatman® Nytran™ N nylon blotting membranes (catalogue: WHA10416196) upon MS medium in the dark at 22 ℃ for 4 days. Following this, the membrane was moved to 1.5 mg/L G418 and 300 mg/L timentin-containing Plich medium, and plates were cultured at 18 ℃ for 4 days in the dark. Plich medium is a minimal medium with low nutritional content containing 0.5 g KH_2_PO_4_, 0.25 g MgSO_4_∙7H_2_O, 1 g asparagine, 1 mg thiamine, 0.5 g yeast extract, 10 mg β-sitosterol, 25 g glucose, and 15 g agar per liter [39]. Further selection was carried out by covering G418-containing rye-sucrose + V8 medium onto the Plich medium twice, and the concentration of G418 were 3 mg/L and 5 mg/L. After approximately 10 days, the regenerated colonies were cut and transferred to 5 mg/L G418-containing rye-sucrose + V8 medium for further screening.

### Bioluminescence detection

Briefly, 1 mM luciferin (catalogue No.7903; Biovision, USA) was evenly sprayed on the surface of mycelium, leaf, tuber and tomato fruit samples. Subsequently, the bioluminescence of transformants and other PiLuc-related samples were detected by Tanon 5200Multi (Shanghai Tanon Life Science Co.,Ltd. China). The images were adjusted and merged by the Tanon Image System (Shanghai Tanon Life Science Co.,Ltd. China). Bioluminescence quantification was defined by bioluminoscore which is quantified by grayscale of images by ImageJ (https://imagej.net/ij/).

### Western blotting

Five-days old *P. infestans* mycelia collected from PEA liquid medium was ground in liquid nitrogen. Then 800 µL lysis buffer (1% SDS in TE buffer) was added to the pulverized mycelium powder and mixed by Vortex-Genie 2 (Scientific Industries, USA) for 30 min at 4 ℃. Supernatants of the protein sample were mixed with loading buffer (Beyotime, P0015) and denatured at 95 ℃ for 10 min. Then, 20 µL samples were loaded into SDS–polyacrylamide gel electrophoresis (SDS-PAGE) and then transferred onto polyvinylidene difluoride (PVDF) membrane by eBlot™ L1 Fast Wet Transfer System (GenScript Biotech Corporation) for detection. The primary antibodies used were, Tag‑DYKDDDDK‑Tag (3B9) Mouse Antibody (Abmart) and H3 (abcam, ab1791), both diluted at 1:5000. Goat-anti-rabbit IRDye 800 CW antibody (Odyssey, no. 926-32211, Li-Cor) was used as secondary antibody, diluted at 1:10000. The signals were detected using the Odyssey laser imaging system (LI-COR company).

### Southern blotting

Southern blotting hybridization was performed according to published protocol [40]. Approximately 30 μg genomic DNA from each line was digested with HindIII and the resulting DNA fragments were separated on a 1% agarose gel using 20 V overnight. Subsequently, the separated fragments were blotted onto a nylon membrane (catalogue: WHA10416196). The blots were probed with a 506 bp PCR fragment amplified using forward primers 5′-GGAGAACTCCCTCCAGTTCTTCA and reverse primer 5′-TGAACATGCCGAAGCCGTGG. This DNA probe was labeled with digoxigenin using DIG-High Prime DNA labeling and detection Starter Kit I (Cat. 11745832910), and was hybridized to the *luc* gene. The blots were probed overnight at 63 °C, washed, and color development was performed according to the standard protocol [40].

### Development phenotypes and pathogenicity of *P. infestans* strains

JH19 and PiLuc strains were grown on rye-sucrose + V8 medium at 18 ℃ in the dark. The diameter of JH19 and PiLuc strains was measured at 6 th days and the average growth rate was calculated as (cm/day). The pathogenicity was performed on detached potato leaves by zoospores following a published method [41]. Sporangia of JH19 and PiLuc strain were harvested from 12-day-old rye-sucrose + V8 medium plates using 4 ℃ pre-cooled water, and the zoospores were collected after incubation at 4 ℃ for 1–2 h and then diluted to 200 zoospores/μL. Three detached potato leaves were placed on wet paper towels and inoculated with approximately 2000 to 4000 zoospores, and then incubated for 4 days at 18 ℃ (14 h light/10 h dark cycle). DNA of 2 cm × 2 cm infected leaf samples was extracted from using the TIANGEN DNA extraction tik (cat. DP304). The biomass of *P. infestans* JH19 and PiLuc infected samples was compared using absolute quantitative PCR (qPCR). Briefly, the DNA concentration of the mycelium sample was measured using Qubit 3.0, and then we prepared the DNA solutions by ten-fold serial dilution. DNA samples of ten-fold serial solutions and infected samples were used as templates in qPCR using the ChamQ Universal SYBR qPCR Master Mix (Vazyme, Cat: Q711-02) following the manufacturer’s instructions. We used Excel to fit a linear regression curve to the DNA concentration and the Ct values obtained. Subsequently, we calculated the DNA concentration of infected samples based on Ct value. These assays were repeated at least 3 times.

### High-throughput screening in 96-well plates

The fungicide and chemical stock solutions were prepared by dimethyl sulfoxide at the concentration of 10^5^ μg/mL and stored in - 20 ℃. Zoospores were collected and diluted into 10 zoospores/μL in PEA liquid medium. Subsequently, 190 μL of zoospore stock and 10 μL different chemicals were loaded into each 96 well plate and the control wells added 190 μL of zoospore stock with 10 μL PEA liquid medium or just ddH_2_O. The final concentration of fluazinam, cyazofamid and cymoxanil were 0.1, 0.01, 0.001 and 0.0001 μg/mL. The final concentration of chemicals was 1 μg/mL. The plates were incubated for 7 days at 18 ℃. The bioluminoscore was detected as described, and the signal density was quantified by ImageJ. The inhibition ratio was defined as (integrated signal density of PiLuc + PEA medium−integrated signal density of chemicals + PEA medium)/integrated signal density of PiLuc + PEA medium. Fluazinam, cyazofamid and cymoxanil were from Suli Co., Ltd. The chemicals (from A1-C21) were kindly provided by the College of Science at Nanjing Agricultural University.

### Potato tuber and tomato fruit infection assay

A syringe needle was used to create 8–10 concentrated holes on the surface of the potato tuber. The wounded potato was then immersed in a zoospore suspension (50 zoospores/μL) for 2 h. The immersed potatoes were incubated at 18 ℃ in the dark for 5 days. After incubation, the infected potatoes were sliced and 1 mM Luciferin was sprayed on the surface of tuber slides. The bioluminescence of tuber slide samples was detected as described above. These assays were repeated 3 times.

For tomato resistance comparison, the pedicel of tomato fruits was removed, and then a syringe needle was used to create 8–10 concentrated holes on the pedicel attachment point. Wounded tomato fruits were then drop-inoculated with 4000 zoospores of PiLuc. Three independent tomato fruits were used. The tomatoes were incubated at 18˚ C in the dark for 3 days. Afterwards, 1 mM Luciferin was sprayed on the surface of the tomato slides. The bioluminescence of tomato samples was detected as described above.

### Evaluation of fungicide bioavailability and fungicide minimum effective dosage in potato tubers

Syringe needles were used to create 8–10 concentrated holes on the surface of potato tubers. The wounded potato was then immersed in a zoospore suspension (50 zoospores/μL) for 2 h. The inoculated potatoes were incubated at 18 ℃ in the dark for 5 days. After incubation, the infected potatoes were cut into four pieces from the wound and confirmed by bioluminescence detection as described above. The potato pieces were immersed in the solutions consisting of 687.5 g/L Fluopicolide·propamocarb Hydrochloride SC (final concentration = 1.375 g/L), 100 g/L Cyazofamid SC (final concentration = 0.1 g/L) and 500 g/L fluazinam SC (final concentration = 0.58 g/L) for 10 min. The potato samples were incubated at 18˚ C in the dark for 6 days, during which bioluminescence data was recorded from 1 to 6 days. The tubers were cut again to assess the bioluminescence within the potato at 6 days post fungicide treatment (dpft).

Potato tubers were soaked into 0, 1.375 × 10^–4^, 1.375 × 10^–3^, 1.375 × 10^–2^, 1.375 × 10^–1^ and 1.375 g/L Fluopicolide ·Propamocarb Hydrochloride SC for 10 min, respectively. Each tuber was cut into four pieces once the surface was dry and then the tubers were inoculated with PiLuc mycelium plugs at 18 ℃ in the dark. The bioluminescence was detected from 1-day post-infection (dpi) to 7 dpi as described above. The 687.5 g/L Fluopicolide·propamocarb Hydrochloride SC, 100 g/L Cyazofamid SC and 500 g/L Fluazinam SC were purchased from Taobao (online shopping platform).

## Results

### Generation of luc-labeled *P. infestans* strain

For pathogen monitoring, we decided to generate a stable luc-labeled *P. infestans* strain. To enhance protein expression, we optimized the firefly *luc* sequence. The codons with the lowest usage probabilities were optimized to relative synonymous codons based on codon bias of *P. infestans*. We optimized 353 codons, and the optimized *luc* and firefly *luc* had 76.29% nucleotide sequence similarity (Fig s1a-d, Table s1).

**Fig. 1:**
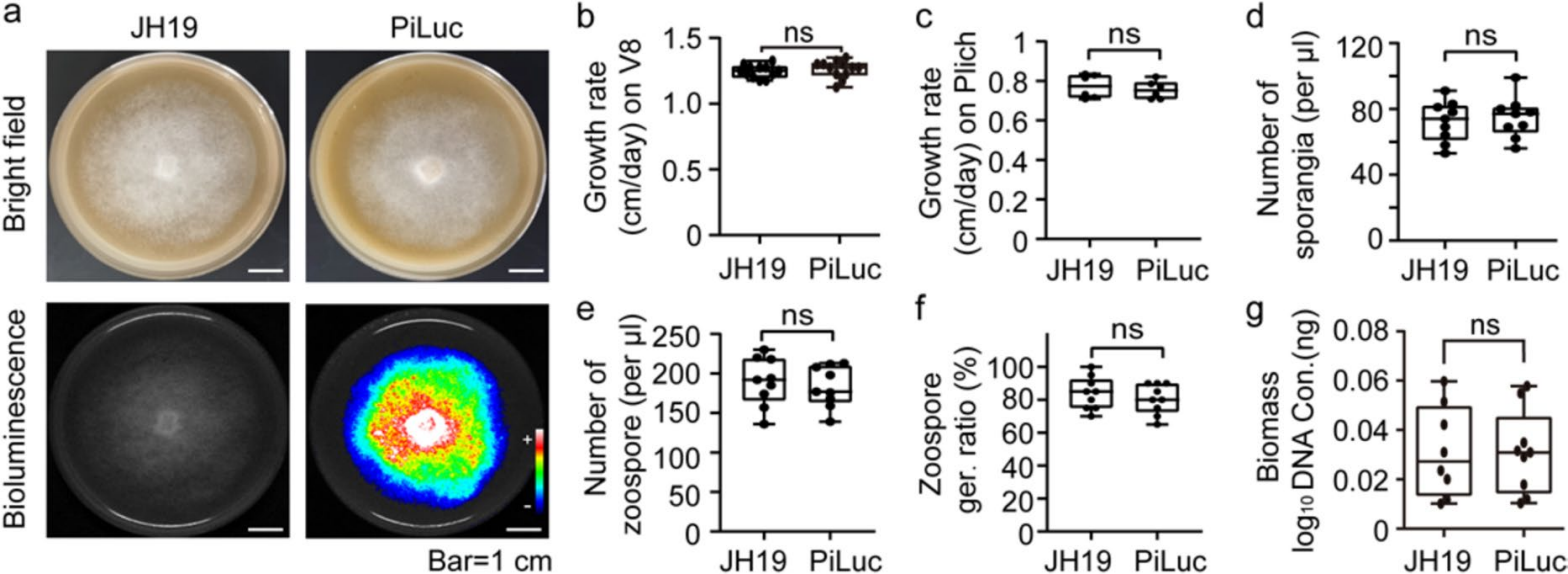
Generation of *luc*-labeled *P. infestans* strain. **a** Morphology and bioluminescence signal of wild type JH19 and luc-labeled strain PiLuc, which were captured at 6 days. + : strong signal; −: weak signal. **b**, **c** Comparison analysis of the growth rate of JH19 and PiLuc on V8 and Plich medium, respectively. Data are shown as the mean ± SD (n = 12). The y-axis is the growth rate (cm/day). **d**–**f** Comparison of sporangia number, zoospore number and zoospore germination ratio of JH19 and PiLuc. The experiments had at least three replicates. **g** Comparison evaluation of pathogenicity between JH19 and PiLuc. Data are presented as mean ± SD (n = 9). The y-axis indicates the biomass (log_10_DNA Con. (ng)) of *P. infestans*. ns indicates no significant difference between JH19 and PiLuc (Students’ t-test)

We constructed a plasmid containing the codon optimized *luc* gene fused with a FLAG tag, which is under the control of promoter of a highly constitutive *Bremia lactucae Ham34* gene [42, 43] (Fig s1e). This plasmid was transformed into *P. infestans* strain JH19 by AMT [37]. Southern blotting result showed multiple copies of *luc* gene were inserted into the genome (Fig s2a). Additionally, both bioluminescent and western blot signal were detected in the transformant, while no signal was detected in the JH19 strain (Fig. 1a, Fig s2). This AMT approach results in a chromosome inserted and genetically stable *luc-expressed P. infestans* strain, allowing manipulation without any antibiotic. Henceforward, the selected transformed strain was named as PiLuc. To rule out changes in growth fitness and infectivity of *luc* inserted strain, we compared the growth, sporangia, zoospore phenotype and infectivity of PiLuc with wild-type strain JH19. There’s no difference in development and infection ability between the two strains (Fig. 1b–g). These results suggest that the expression of *luc* on PiLuc does not alter the growth or infectivity of *P. infestans* under lab conditions. Besides, there’s no difference in expression levels among mycelium, sporangium, zoospore stages and 1–3 dpi (Fig s3). This suggests that the optimized *luc* gene was continuous expressed in *P. infestans*.

### High-throughput screening of chemical fungicides in 96-well plate format

**Fig. 2:**
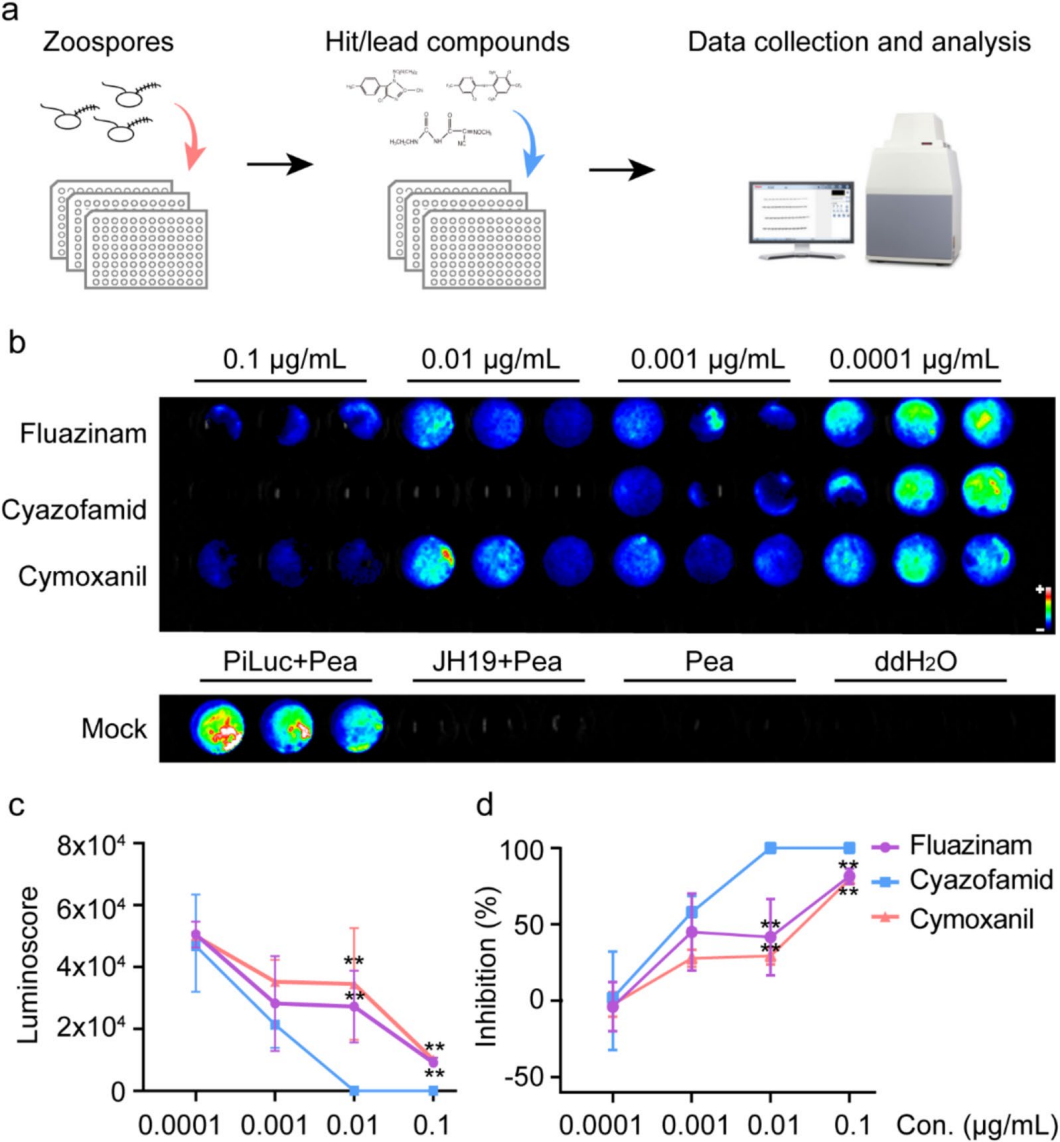
High throughput screening of chemical fungicides in 96-well format. **a** The schematic representation of the high throughput screening in 96-well format. **b** The bioluminescence result of 96-well plate inhibition assay at day 7. First three lanes display the bioluminescence outcomes of fluazinam, cyazofamid and cymoxanil in four concentrations. The mock lane shows the bioluminescence result of PiLuc + Pea medium, JH19 + Pea medium, Pea medium and ddH2O. **c** and **d** Line chart depicting bioluminoscore and inhibition rates of fluazinam, cyazofamid and cymoxanil in four concentrations. X-axis represents fungicides concentration; the y-axis indicates the inhibition rate (%) showing the means ± standard deviation (SD) (n = 3 biological replicates). The inhibition rate was calculated as (integrated signal density PiLuc + PEA medium- integrated signal density chemicals + PEA medium)/integrated signal density PiLuc + PEA medium

Drug discovery increasingly relies on high throughput technologies to efficiently screen chemical libraries. We compared the inhibition properties of fluazinam, cyazofamid and cymoxanil by conducting a serial dilution of chemicals (Fig. 2a, b). Firstly, no signal was detected in the JH19 + Pea medium, Pea medium only or ddH_2_O only treatment, suggesting that the bioluminescent signal was emitted by *luc*-labeled strains. The bioluminescence and bioluminoscore both increased with the serial dilution of three fungicides, implying the growth of PiLuc was inhibited by the three chosen fungicides in a dosage-dependent manner. No signal was detected in 0.1 μg/mL and 0.01 μg/mL of cyazofamid treatment, suggesting that zoospores were totally inhibited in these two concentrations (Fig. 2b). Additionally, we can easily conclude that the zoospore inhibition property of cyazofamid is significantly higher than the other two fungicides under laboratory condition (**P < 0.01; one-way ANOVA) (Fig. 2c, d).

To further explore the utility of PiLuc in 96-well plate format, we used it to compare the inhibition ratio of three sets of published compounds (Fig s4). Using this approach, we observed a tenfold range of bioluminescent signals in the plate, and the inhibition ratio ranged from 0 to 47% (Fig s4). In summary, our approach could be used for rapid, large-scale quantification of the inhibition properties of chemicals.

### Monitoring of early infected area by PiLuc

**Fig. 3:**
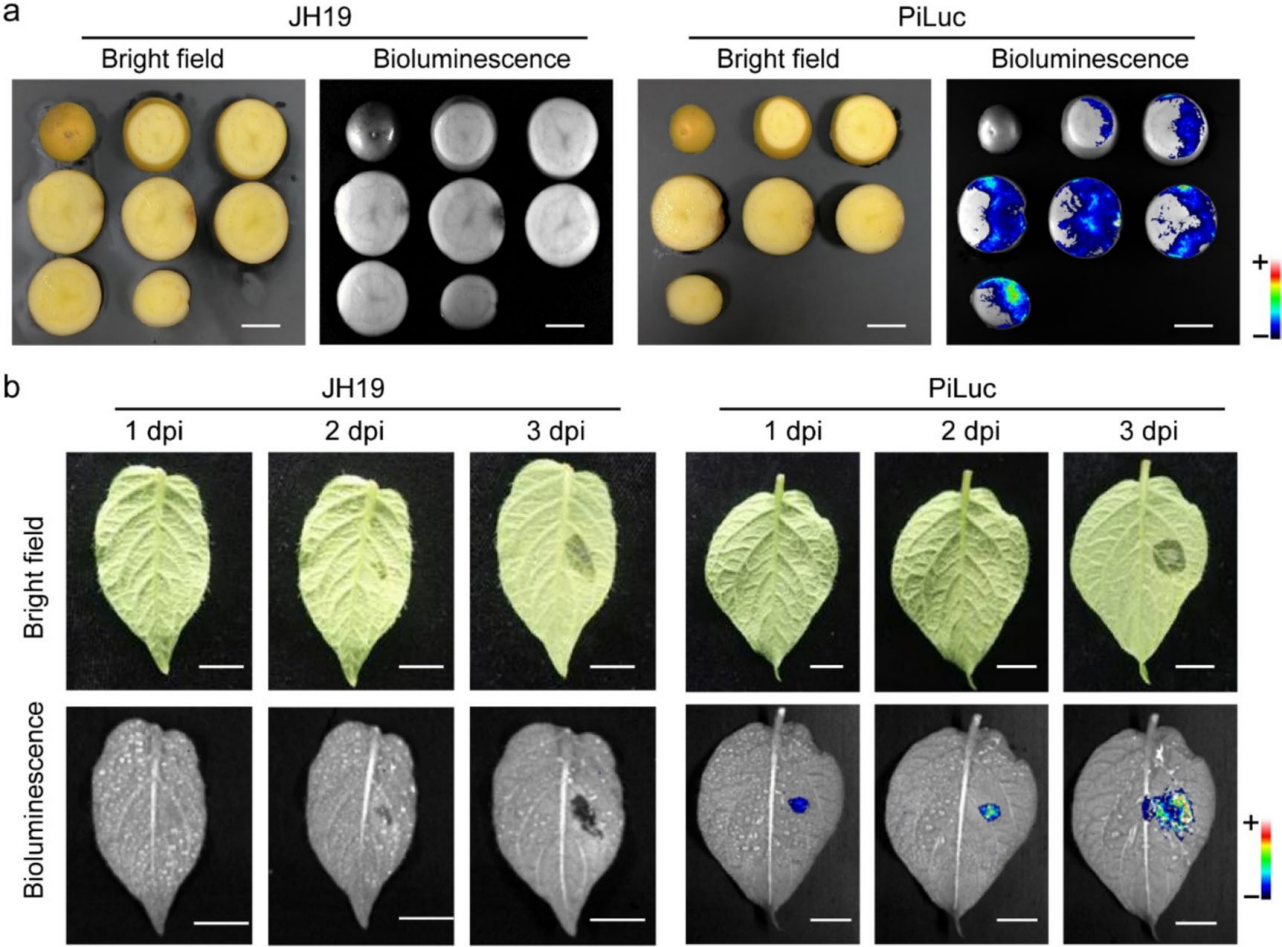
Monitoring of early infected area by PiLuc on potato tubers and leaves. **a** Bright field and bioluminescence of potato slides inoculated with PiLuc at 7 dpi. **b** Bright field and bioluminescence of potato leaves inoculated with PiLuc from 1 to 3 dpi. + : strong signal; −: weak signal

To evaluate the suitability of PiLuc for monitoring *P. infestans* infection dynamics during colonization process, we conducted infection assays through inoculation of potato tubers and tomato fruits with PiLuc zoospore suspensions. Notably, bioluminescence imaging revealed unexpectedly extensive infection areas, particularly in certain tuber tissues that remained macroscopically asymptomatic. The strains propagated obviously from the vascular ring (Fig. 3). We further challenged fruits from two tomato cultivars (*Solanum lycopersicum* cv. *Qianxi* and cv. *green cherry*) with PiLuc zoospores. Notably, both cultivars lacked discernible necrotic lesions during the observation period, yet bioluminescence imaging revealed detectable pathogen colonization in all inoculated samples. The comparative different bioluminescence areas demonstrated differential susceptibility, with *Qianxi* tomato fruits exhibiting higher colonization levels than *green cherry* (Fig s5). The enhanced spatiotemporal resolution of PiLuc-mediated monitoring prompted systematic investigation of early infection on leaves, given that late blight is an airborne plant disease and most researches focus on leaf infection. Bioluminescent signals were detectable in PiLuc-inoculated detached leaves as early as 2 dpi, while the bright field picture of PiLuc infected samples revealed no discernible symptomatic lesions at the same time (Fig. 3b). Collectively, PiLuc can track the colonization dynamics across multiple host tissues-including potato leaves, tubers and tomato fruits with articular sensitivity for detecting early infection stages and conventional visual assessment-invisible infection areas.

### Quantitative detection of *P. infestans* biomass by bioluminescence in plant-pathogen interaction

**Fig. 4:**
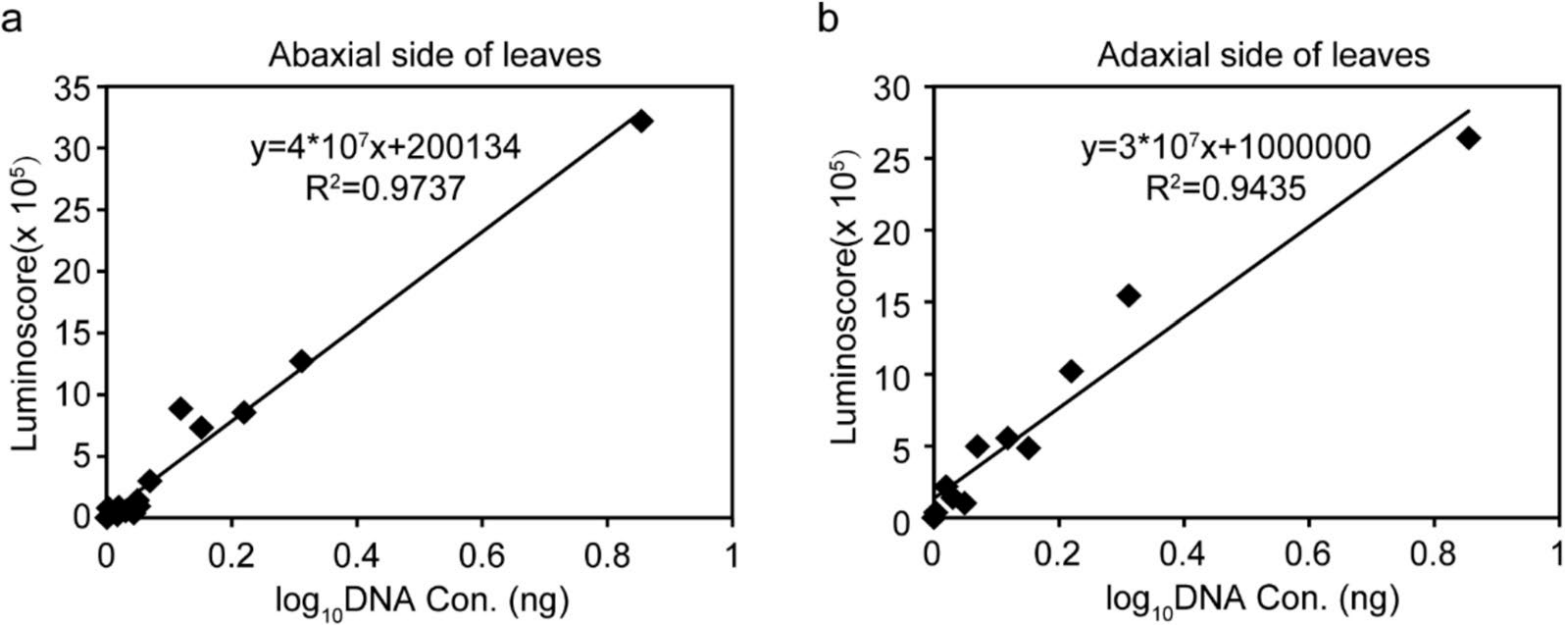
The luminoscore of PiLuc is linearly correlated to its concentration in leaf infection assay. **a**, **b** Correlation between DNA concentration and luminoscore on the abaxial side and the adaxial side, respectively. X-axis represents the DNA concentration, y-axis indicates luminoscore

To establish the quantitative relationship between bioluminescent signal of PiLuc and pathogen biomass during infection stages, we conducted correlative quantification of the bioluminescent signal and relative biomass of infection samples. In foliar infection models, it revealed strong linear correlations (R^2^ = 0.9737 [abaxial]; R^2^ = 0.9435 [adaxial]) between bioluminescent signal and biomass, respectively indicating strong correlations between the bioluminescent signal and biomass (Fig. 4). It indicates that the bioluminescent signal had a linear logarithmic relationship with pathogen biomass in the leaf infection assay. To further validate the effectiveness of our method, we prepared zoospore-infected potato tuber samples. Contrastingly, three-dimensional tuber infection assays showed statistically insignificant correlation (R^2^ = 0.2955), likely due to signal attenuation in parenchymatous tissues (3.2 ± 0.5 mm penetration depth vs. 0.1 mm leaf thickness). These results validate that PiLuc as a reliable real-time biomass proxy specifically in leaf infection, while highlighting detection limitations in geometrically complex host tissues.

### Evaluation of fungicide on potato tubers

**Fig. 5:**
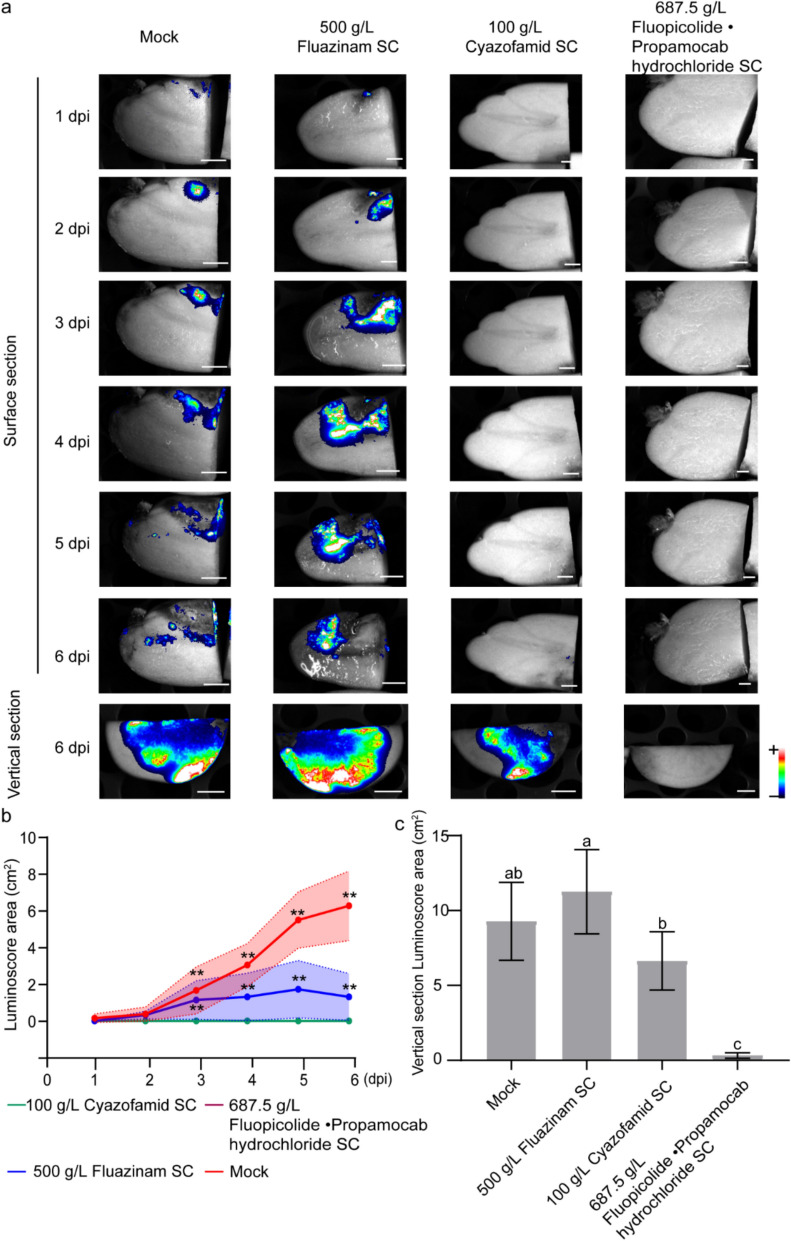
Evaluation of fungicide bioavailability in potato tubers. **a** The bioluminescence results of surface and vertical section from pre-treated potato tubers with 687.5 g/L Fluopicolide·propamocarb Hydrochloride SC (1.375 g/L),100 g/L Cyazofamid SC (0.1 g/L) and 500 g/L fluazinam SC (0.58 g/L) were evaluated from 1 to 6 dpft. **b** Line chart of bioluminoscore area from surface of pre-infected potato tubers with four fungicides from 1 to 6 dpft. Y-axis indicates luminoscore area (cm^2^). Different lines represent luminoscore area of four fungicide treatments from four replicates. Asterisks denote significant differences (**P < 0.01; one-way ANOVA across treatment at each individual time-point). **c** Comparison of luminoscore area (cm^2^) within potato among four different treatments at 6 dpft. Different letters indicate significant differences (**P < 0.01; one-way ANOVA). Experiments were conducted at least three times with similar results

Capitalizing on the non-invasive monitoring capacity of the PiLuc in tuber-pathogen interactions, we developed a fungicide evaluation platform optimized for tubers. The model integrates real-time bioluminescent quantification with fungicide mobility profiling, assessing both contact and systemic compounds. Pre-infected tubers were incubated with fungicides followed by 1–6 days continuous bioluminescence monitoring (Fig. 5). Among the tested fungicides, 100 g/L cyazofamid SC and 687.5 g/L Fluopicolide·Propamocab hydrochloride SC significantly inhibited the bioluminescence of PiLuc on the surface of potato tubers compared with mock treatment (Fig. 5a). To characterize fungicide translocation efficiency, we employed bioluminescence tomography to map transport of candidate compounds. Notably, no luminescence was observed in the tubers treated with 687.5 g/L Fluopicolide·Propamocab hydrochloride SC, indicating successful uptake of the fungicide and inhibition of *P. infestans* spreading inside the tubers (Fig. 5a). Notably, tubers subjected to 100 g/L cyazofamid SC treatment exhibited bioluminescence inside the tuber, indicating effective surface pathogen suppression but incomplete eradication of pre-established infection (Fig. 5a–c).

**Fig. 6:**
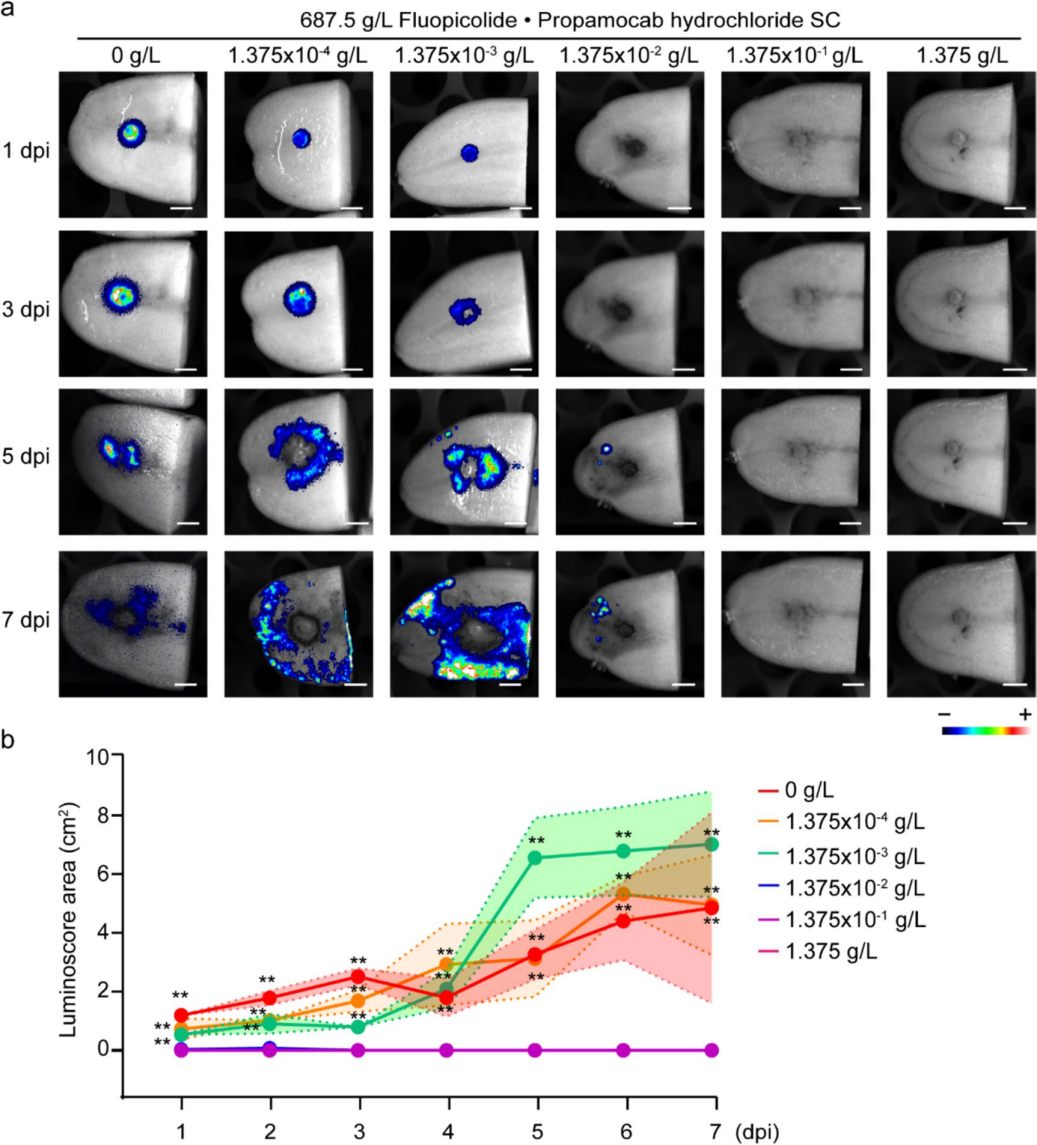
Evaluation of fungicide minimum effective dosage in potato tubers. **a** The bioluminescence results of pre-treated potato tubers with 687.5 g/L Fluopicolide·Propamocab hydrochloride SC were evaluated at 1, 3, 5, and 7 dpi. **b** Line chart of bioluminoscore area of pre-treated potato tubers with 687.5 g/L Fluopicolide·Propamocab hydrochloride SC. Y-axis represents luminoscore area (cm^2^). Different lines represent luminoscore area of six different concentrations of fungicide treatments from four replicates. Asterisks denote significant differences (**P < 0.01; one-way ANOVA across treatment at each individual time-point). Experiments were conducted three times with similar results

We conducted prophylactic treatment of tubers using logarithmic fungicide gradients (1.375, 1.375 × 10^–1^ and 1.375 × 10^–2^ g/L), followed by zoospore inoculation. Quantitative analysis demonstrated dose-dependent inhibition efficacy, with the 1.375 × 10⁻^1^g/L Fluopicolide·Propamocab hydrochloride SC achieving complete suppression of *Phytophthora infestans* zoospore colonization throughout the 7-day monitoring period (Fig. 6a, b). This protocol enables standardized evaluation of fungicides activity for potato tuber protection.

## Discussion

Visualization and quantification of pathogens are essential for optimizing fungicide development and deployment. However, current methods have certain limitations, underscoring the necessity for a convenient and efficient method to visualize and quantify pathogen colonization, particularly for *P. infestans* [12]. In this study, we developed a *luc*-labeled *P. infestans*, enabling the establishment of an imaging technology-based fungicide screening method. This method enables precise visualization of pathogens and straightforward quantification in both 96-well plates and host plants.

Codon usage can restrict the applicability of naturally occurring reporter genes to certain species. To address this, we transformed an optimized firefly *luc* gene into *P. infestans* under a constitutive promoter (Fig. 1, Fig s1, Fig s2). Three out of 43 (7.0%) independent colonies were positive transformants (data not shown), demonstrating that the *luc*-optimized vector’s capacity to serve as an efficient reporter system. This result aligns with the hypotheses proposed in prior studies [35]. Although multiple T-DNA insertions in PiLuc, *luc* gene expression remained consistent across mycelium, sporangium, zoospore and 1–3 dpi stages (Fig s2, Fig s3). This suggests the overall expression pattern is relatively stable among PiLuc development stages. Codon optimization increases translational efficiency and protein expression without altering protein sequence [44–46]. This approach is particularly useful in heterologous expression proteins [47, 48]. We speculate that the optimized *luc* gene sequence could be valuable not only in visualizing microbes but also for studying protein–protein interaction and gene expression profiling in oomycete research.

96-well plate screening method reduces experimental time and labor requirements while providing a cost-effective approach for quantitative assessment of inhibition activity of compounds in zoospore-based in vitro models. This facilitates screening of compounds suppressing zoospore germination and mycelium growth (Fig. 2, Fig s4). The use of zoospores, sporangia, and mycelial fragments as inocula enables evaluation of chemical inhibitory efficiency across multiple developmental stages. Nonlinear regression analysis for EC_50_ determination using agar medium-based assays remains the standard methodology for assessing fungicide sensitivity in oomycetes. The determination of the inhibition ratio of three fungicides by the standard assay required more than fifty 7 cm petri dishes and a solid medium. In contrast, our method obtained the equivalent data with only one 96-well plate in about 1 h, including sample loading and data collection. This approach reduces the experimental duration compared to conventional in vitro assays. Additionally, only 200 μL liquid PEA medium was loaded into each well of the 96-well plate, we conclude that this protocol is also suitable for those “infrequent” chemicals, including low-content natural products and low-yield chemicals. Our comparison result matched the reported EC_50_ value of these three fungicides with published data [49–51]. The high-throughput pathogen capture capacity enables efficient compound prioritization within chemical discovery pipelines. Future integration of automated liquid handling platform with CCD/CMOS camera and automated image analysis algorithms may enhance screening throughput and analytical precision.

Fluorescent protein (green, red, and so on) labeled strains have become a standard method for studying plant-pathogen interaction, and are particularly advantageous for high magnification microscopic studies [52–54]. However, their broader application is constrained by the limited observation scale and the high cost of instruments. Additionally, the spontaneous ultraweak light emission from plants can interfere with microscopic observations, complicating the detection of specific fluorescent signals emitted by labeled pathogens. No bioluminescent signal was detected even at late infection stage in wild-type strain-infected sample (data not shown), suggesting the bioluminescent signal specificity of *luc*-labeled strains. The application of bioluminescence imaging has significantly enhanced our understanding of infection processes at a larger observation scale. While typical symptoms of late blight have been well-documented in both educational resources and published papers, the visible assessment of symptomatic areas remains subject to observer variability [35]. In this study, we documented the late blight infection process observing that infected regions were notably larger compared to the lesion areas on both leaves and tubers (Fig. 3). Notably, the vascular ring demonstrated high susceptibility to infection, with lesions exhibiting irregular spread patterns into the cortex and perimedullary zone (Fig. 3a). These observations provide valuable data for phenomics research, particularly since the process allows for real-time imaging of individual plants throughout experimental periods.

This study enables the quantitative analysis of *P. infestans* in infected leaf samples. While bioluminescence has been widely applied in plant-bacteria pathogen systems for quantitatively comparison of infection processes, its application in plant-filamentous pathogen systems remains limited [22, 55]. By employing a leaf infection assay, we can efficiently evaluate the resistance levels of different cultivars, as the bioluminescent signal exhibits a linear logarithmic correlation with pathogen biomass in leaf samples (Fig. 4). This approach significantly reduces the time, reagent, and consumable costs compared to alternative methods such as real-time PCR. Additionally, we developed a semi-non-destructive method for collecting time-course data in leaf infection assay, which minimizes experimental error by enabling continuous monitoring of the same sample. This method is expected to prove valuable for physiological studies of plant-pathogen interaction mechanisms, particularly in the leaf system. However, it is unsuitable for quantifying the pathogen biomass in tuber infection samples. The limitation is likely due to the poor luciferin substrate uptake in certain tissue, such as tubers, and the restricted contact of infected mycelium with substrate caused by the complex three-dimensional structure of tuber infections. Further investigation into appropriate tuber sample preparation techniques, including the use of a microtome, is warranted.

Our study validates PiLuc as an effective tool for assessing chemicals bioavailability. Previously, techniques such as radioactive tracing, fluorescent labeling technique and HPLC–MS have been predominantly employed for evaluating pesticides absorption [56–58]. The established protocol utilizes bioluminescent signal as an indicator of fungicide bioavailability, offering a more cost-effective and user-friendly system compared with existing methods. Fluopicolide·Propamocarb hydrochloride SC effectively protects tubers from PiLuc infection in lab conditions (Figs. 5 and 6), while cyazofamid SC effectively eliminated PiLuc on the potato tuber surface, significant internal infection persisted (Fig. 5).

## Conclusion

We have successfully generated a *luc*-labeled *P. infestans*, enabling the establishment of an imaging technology-based fungicide screening method. This method facilitates precise pathogens visualization and enables efficient quantification in both 96-well plates and host plants. In summary, we have developed a user-friendly bioluminescent imaging system for quantitative evaluation of fungicide candidates and in planta pathogen visualization.

## Supplementary Information

Supplementary Material 1.

Supplementary Material 2.

## Data Availability

No datasets were generated or analysed during the current study.

## Abbreviations

PCR: Polymerase chain reaction (PCR)
IF: Immunofluorescence
ELISA: Enzyme-linked immunosorbent assay
MS: Mass spectrometry
luc: Luciferase
PLB: Potato late blight
AMT: Agrobacterium mediated transformation
SDS-PAGE: SDS-polyacrylamide gel electrophoresis
PVDF: Polyvinylidene difluoride
qPCR: Quantitative PCR
