# Supplementary material for: Bioluminescent imaging of an oomycete pathogen empowers chemical selections and rational fungicide applications
Source: Plant Methods. 2025 May 7;21:57. doi: 10.1186/s13007-025-01374-9 (PMC12060300; doi:10.1186/s13007-025-01374-9)
Supplement: Supplementary file 1 — Supplementary Material 1. [file 13007_2025_1374_MOESM1_ESM.docx]

**Supporting information**

*Bioluminescent imaging of an oomycete pathogen empowers chemical selections and rational fungicide applications*

Fig s1 Schematic diagram of vector and sequence of optimized luciferase gene for transformation

(a), Sequence of optimized luciferase gene. (b)-(d), Comparison the codon usage of arginine, serine and threonine among *P. infestans* 325 CDSs, optimized sequence and original sequence. (e), Schematic diagram of vector for *P. infestans* AMT.

Fig s2 Verification of *luc*-labeled *P. infestans* strain PiLuc by southern blotting and western blotting

(a), southern blotting result of JH19, PiLuc and mock (ddH_2_O), size of DNA ladders was marked. (b), western blotting result of PiLuc, JH19, CK (the transformed strain harbors G418 resistance but don’t express luc-flag fusion protein) and Turbo-flag (*P. sojae* strain expresses Turbo-flag fusion protein). Size of protein makers was marked.

Fig s3 Comparison of relative expression of *luc* gene among development and infection stages in PiLuc.

X-axis represents different development and infection stages. MY, SP, ZO indicate mycelium, sporangium, zoospore stages. 1 dpi, 2 dpi, 3 dpi indicate 1-3 days post incubation. Y-axis represents relative expression level of *luc* gene compared with mycelium stages. ns indicates no significant difference among different stages (P>0.05, one-way ANOVA).

Fig s4 Comparison of inhibition rate of 56 compounds

(a) and (d), The bioluminescence result of 96-well plate inhibition assay at 7th day. (b) and (e), Sample information of 96-well plates, blue dash line divided the three replicates of each treatment. (c) and (f), Comparison the inhibition rate of different chemicals. The y-axis is inhibition rate (%). The inhibition rate was defined as (integrated signal density PiLuc+PEA medium - integrated signal density chemicals+PEA medium)/ integrated signal density PiLuc+PEA medium.

Fig s5 Late blight resistance comparison of *green cherry* and *Qianxi* tomato fruits.

Bright field and bioluminescence signal of tomato fruit infected by PiLuc, which were captured at 3 days. +: strong signal; -: weak signal. The first lane is *green cherry* tomato; the second lane is *Qianxi* tomato.


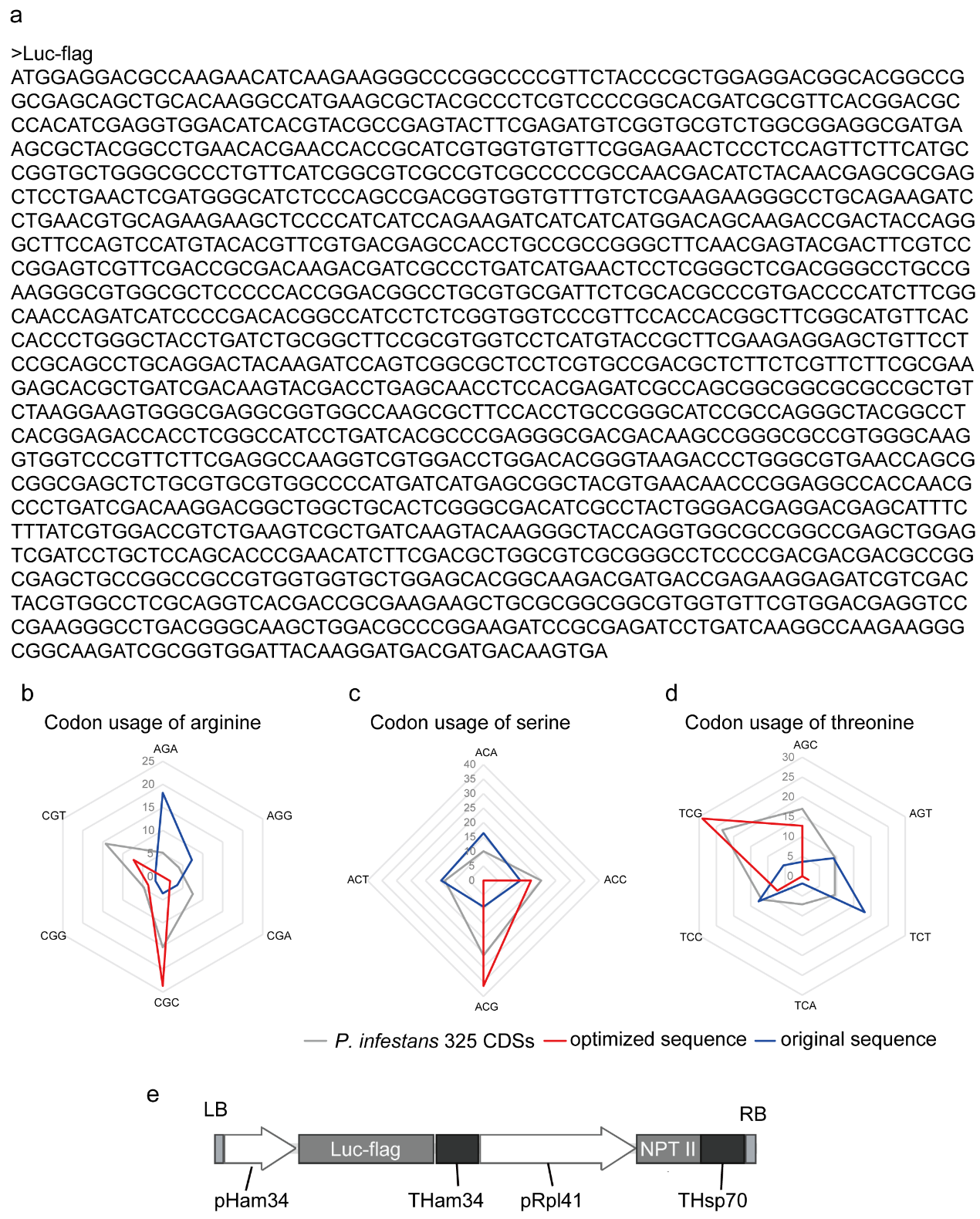
 Fig s1 Schematic diagram of vector and sequence of optimized luciferase gene for transformation





Fig s2 Verification of *luc*-labeled *P. infestans* strain PiLuc by western blot





Fig s3 Comparison of relative expression of *luc* gene among development and infection stages in PiLuc


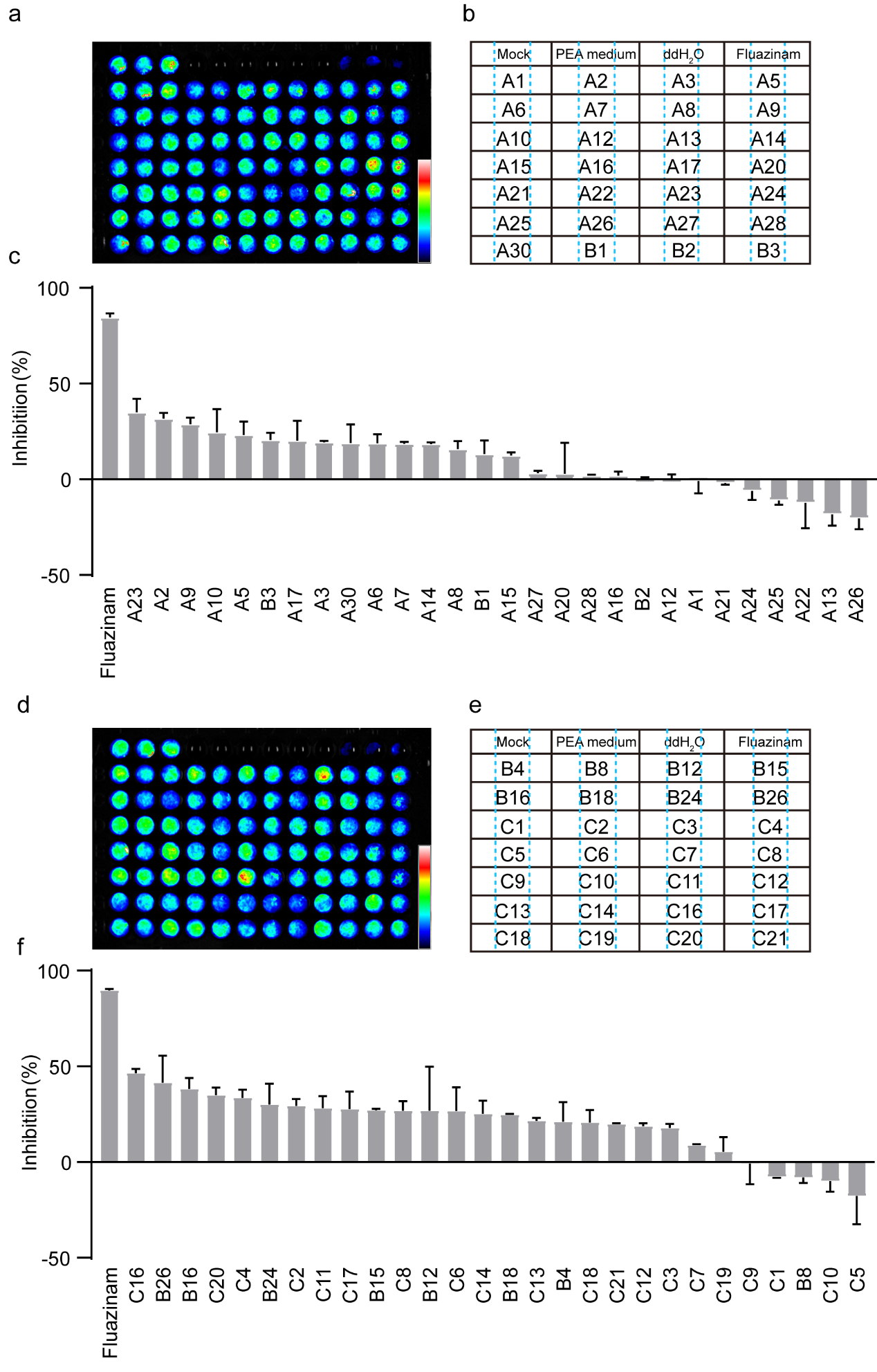


Fig s4 Comparison of inhibition rate of 56 compounds


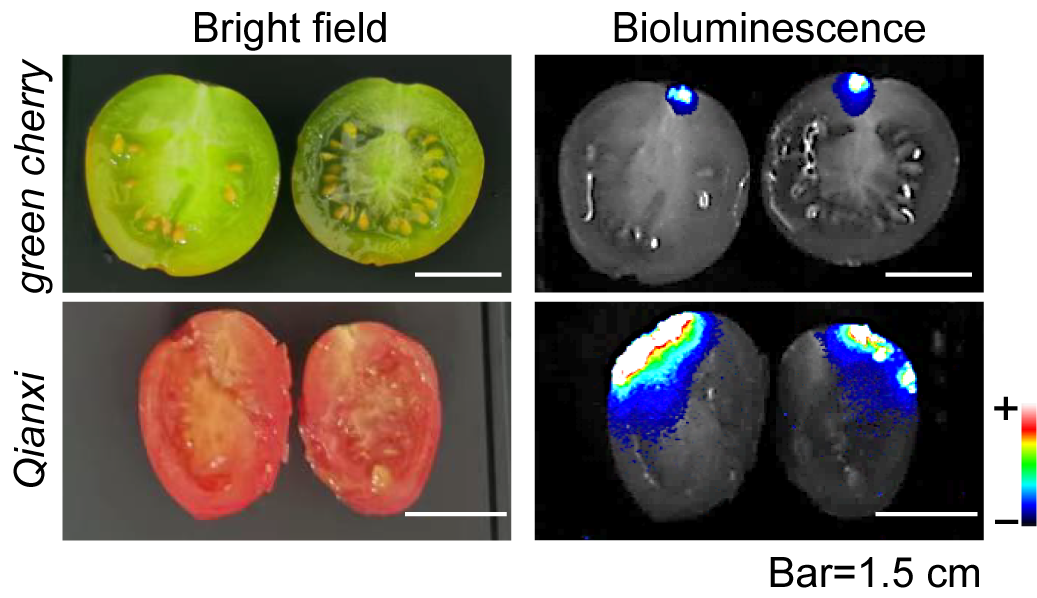


Fig s5 Late blight resistance comparison of *green cherry* and *Qianxi* tomato fruits
